# Potential of guggulsterone, a farnesoid X receptor antagonist, in the prevention and treatment of cancer

**DOI:** 10.37349/etat.2020.00019

**Published:** 2020-10-30

**Authors:** Sosmitha Girisa, Dey Parama, Choudhary Harsha, Kishore Banik, Ajaikumar B. Kunnumakkara

**Affiliations:** Cancer Biology Laboratory and DBT-AIST International Center for Translational and Environmental Research (DAICENTER), Department of Biosciences and Bioengineering, Indian Institute of Technology Guwahati, Guwahati, Assam 781039, India; National University of Singapore, Singapore

**Keywords:** Cancer, *Commiphora wightii*, guggulsterone, Z and E isomers, anticancer activities

## Abstract

Cancer is one of the most dreadful diseases in the world with a mortality of 9.6 million annually. Despite the advances in diagnosis and treatment during the last couple of decades, it still remains a serious concern due to the limitations associated with currently available cancer management strategies. Therefore, alternative strategies are highly required to overcome these glitches. The importance of medicinal plants as primary healthcare has been well-known from time immemorial against various human diseases, including cancer. *Commiphora wightii* that belongs to Burseraceae family is one such plant which has been used to cure various ailments in traditional systems of medicine. This plant has diverse pharmacological properties such as antioxidant, antibacterial, antimutagenic, and antitumor which mostly owes to the presence of its active compound guggulsterone (GS) that exists in the form of Z- and E-isomers. Mounting evidence suggests that this compound has promising anticancer activities and was shown to suppress several cancer signaling pathways such as NF-κB/ERK/MAPK/AKT/STAT and modulate the expression of numerous signaling molecules such as the farnesoid X receptor, cyclin D1, survivin, caspases, HIF-1α, MMP-9, EMT proteins, tumor suppressor proteins, angiogenic proteins, and apoptotic proteins. The current review is an attempt to summarize the biological activities and diverse anticancer activities (both *in vitro* and *in vivo*) of the compound GS and its derivatives, along with its associated mechanism against various cancers.

## Introduction

Cancer is one of the most life-threatening diseases of the present century, which consists of over 277 different types [1]. According to GLOBOCAN 2018, cancer has a very high rate of occurrence with a mortality of around 9.6 million per year globally [2–6]. It is now well-established that the alterations of various vital genes and proteins drive the transformation of normal cells to cancer phenotypes, which ultimately lead to cancer [1, 7–16]. Besides, many factors contribute to the pathogenesis of cancer, including elevated metabolic requirements that lead to the upregulation of enzymes required for the synthesis of fatty acids and modulation of several signaling pathways [17, 18]. These molecular alterations are caused by various factors such as an imbalanced diet, physical inactivity, pollution, and consumption of addictive substances such as tobacco and alcohol [4, 19]. The past couple of decades have evidenced substantial improvements in conventional methods for the treatment of cancer, such as surgery, radiation, and chemotherapy [20–23]. In addition, novel therapeutic modalities have emerged for the treatment of this disease such as immunotherapy, gene therapy, targeted therapy, personalized medicine, and nano vaccines [24–29].

Regardless of the advancement, the treatment approaches have not shown significant improvement in terms of survival and quality of life (QOL) of cancer patients due to several factors such as chemoresistance, radioresistance, adverse side effects of drugs, and cancer recurrence [30–34]. Further, the majority of the drugs used in conventional methods target a single protein or pathway, which induce several survival signals that restrict their efficacy. Therefore, the development of alternative therapy is required for improving the survival and QOL of cancer patients. This drives the urge to develop safe, efficacious, affordable, and multi-targeted agents for the management of this disease [4, 9, 35–38].

Accumulating pieces of evidence, since the rise of human civilization, suggest that medicinal plants have gained colossal importance in different traditional medicinal systems such as Ayurveda, Siddha, Unani, and Traditional Chinese Medicine (TCM) owing to their limitless properties in disease prevention and treatment [5, 39–42]. Studies have also advocated the immense biological properties of compounds isolated from these plants as potential candidates against various fatal diseases, including cancer [43–56]. Guggulsterone (GS) is one of those naturally derived multi–targeted compounds that have exhibited massive therapeutic potential against cancer. Therefore, the current review summarizes its prospects in the prevention and treatment of cancer.

GS is a sterol compound derived from the gum resins of the guggul plant *Commiphora wightii* (*C. wightii*) that belongs to the family of Burseraceae [57, 58]. *C. wightii* is commonly found in Somalia, Northeast Africa, Southern Arab countries, and countries of Southeast Asia such as India, Bangladesh, and Pakistan [59–61]. In India, *C. wightii* is mostly distributed in the States of Maharashtra, Gujarat, Rajasthan, and Karnataka; however, the States of Rajasthan and Gujarat form the commercial centers of this gum [59]. Before being justified with its present name as *C. wightii* by Bhandari, it was named as *C. mukul* or *Blasmodendron mukul* and then as *C. roxburghii* [62]. The oleogum resin of this plant is commonly known as guggul (in Hindi), guggulu (in Sanskrit), gukkulu and maishakshi (in Tamil), and Indian bdellium (in English) [59]. The therapeutic benefits of *C. wightii* have been reported in the treatment of various diseases including tumors, malignant sores, ulcers, obesity, and liver and intestinal problems; hence, it has been widely used in Ayurveda for thousands of years [57]. Besides, GS has also gained importance due to its chemopreventive and therapeutic properties against various cancers and their hallmarks [63, 64]. This plant metabolite was first recognized as an antagonist for the nuclear receptor, farnesoid X receptor (FXR) [57]. The later studies revealed that it could also target the receptors of mineralocorticoid, glucocorticoid, androgen and estrogen [60]. Subsequently, the underlying mechanism behind GS-mediated hypolipidemic effect was identified. GS was found to decrease FXR activity and increase bile salt export pump, a transporter that regulates bile efflux [60]. Further, this sterol was also found to modulate the expression of several proteins such as cyclin D1, c-Myc, matrix metalloproteinase (MMP)-9, cyclooxygenase 2 (COX-2), and vascular endothelial growth factor (VEGF); and anti-apoptotic proteins such as inhibitor of apoptosis protein 1 (IAP1), X-linked inhibitor of apoptosis protein (XIAP), B-cell lymphoma 2 (Bcl-2), Bcl-2-related protein A1 (Bfl-1/A1), cellular FLICE (FADD-like IL-1β-converting enzyme)-inhibitory protein (cFLIP), and survivin in various cancer models [64]. GS has also shown to suppress tumor necrosis factor (TNF)-α, IkappaB kinase (IKK), IkappaB alpha (IκBα), nuclear factor kappa B (NF-κB) activation, and NF-κB regulated gene expression [64, 65]. This compound was also reported to modulate other pathways like phosphoinositide 3-kinase (PI3K)-Akt, steroid receptor coactivator (Src)/focal adhesion kinase (FAK), Janus kinase (JAK)/signal transducers and activators of transcription (STAT) and their downstream molecules [66, 67].

As mentioned, GS acts as an antagonist for FXR. Studies have reported that FXR helps in stabilizing the metabolism of bile acids and cholesterol and it is involved in the development of different diseases such as cardiovascular diseases and cancer [57, 68, 69]. Numerous lines of evidence also suggest that interruption of cholesterol homeostasis is one of the major events in cancer development as the failure in the sustenance of cholesterol synthesis through feedback inhibition results in high recruitment of cholesterol and its precursors in cancer cells [70, 71]. Thus, GS, being an FXR antagonist, could be used as an effective regimen for the treatment of cancer. Therefore, through inhibition of FXR and modulation of several other genes and proteins, GS has shown promising effects in the prevention and treatment of different cancers.

## Sources of GS

GS is the main component of guggulipid, which consists of a mixture of diterpenes, steroids, sterols, esters, and higher alcohols, extracted from the gum resin of the medicinal plant *C. wightii* [72–75]. The Z- and E-stereoisomers of GS represent the main active compound of the plant [76]. The plant gum resin also contains 0.4% essential oils that mainly include myrcene, long-chain aliphatic tetrols that are esterified at the primary-OH group with the ferulic acid [77].

## Traditional uses of *C. wightii*

The importance of *C. wightii* has been mentioned in its ancient medicinal writings of Ayurveda. The Sushruta Samhita explains the use of this plant and its gum resin against various diseases [58]. These medicinal writings mention the oral consumption of guggul can be used to heal conditions such as internal tumors and malignant sores, intestinal worms, liver dysfunction, edema, and to treat inflammatory diseases, gynaecological diseases, and obesity [78, 79]. *C. wightii* is one of the common ancient medicinal plants, that is taken to improve heart condition, vascular health, wound healing, and to treat vitiligo in Ayurveda [80, 81]. Further, the gum resin of *C. wightii* finds its use in TCM for the treatment of arthritis, trauma, and other blood-related diseases [78]. The guggul is also used in Yunani medicine for treating nervous diseases, scrofulous infections, urinary disorders, and skin diseases. It is also locally applied as a paste in hemorrhoids, incipient abscesses, and ulcers [82].

## Biological properties of *C. wightii*

**Figure 1.**
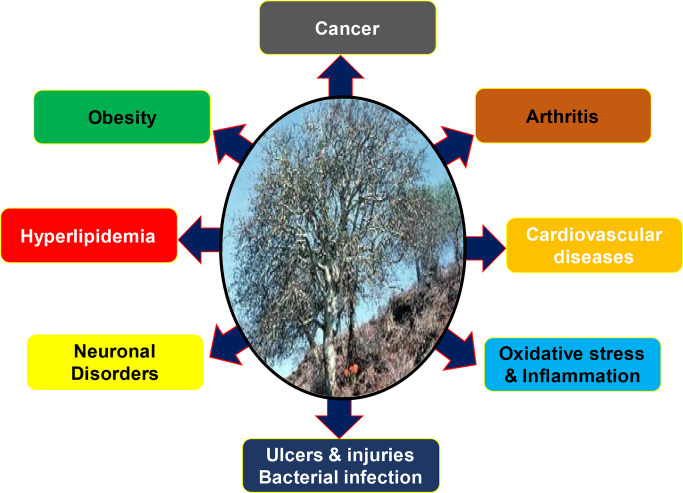
Biological activities of *Commiphora wightii* (Mark W. Skinner/www.discoverlife.org)

It is already well established that the gum resin of *C. wightii* has been used traditionally for centuries against various chronic diseases like arthritis, obesity, diabetes and cardiovascular diseases (Figure 1) [83]. Recent studies have reported that the resin extracts from *C. wightii* lower the levels of low-density lipoprotein (LDL) and cholesterol in different experimental settings [58, 84, 85]. The *C. wightii* was shown to decrease LDL, very-low-density lipoprotein (VLDL), and cholesterol and increase high-density lipoprotein (HDL). It was also shown to reduce high fat-induced obesity in rat models [86]. Additionally, *C. wightii* was reported to improve oxidative stress, inflammation, edema, and necrosis in an ischemic rat model, thereby displaying its cardioprotective effects [80]. In another study, *C. wightii* was also shown to prevent lipid layer damage by inhibiting lipid peroxidation [87]. Further, this plant was shown to inhibit diabetes by decreasing the expression of aspartate aminotransferase, alanine aminotransferase, and oxidative markers, i.e. lipid peroxidation and protein oxidation; and inducing nuclear receptors such as peroxisome proliferator-activated receptor alpha (PPARα), peroxisome proliferator-activated receptor-gamma (PPARγ) and liver X receptor [88, 89]. Besides, another constituent, dehydroabietic acid, from *Commiphora sp.* was found to enhance the diabetic wound healing via reversing TNF-α-induced activation of forkhead box protein O1 (FOXO1) and the transforming growth factor β (TGF-β)1/Smad3 signaling [90].

Further, guggulipid could act as an antinociceptive agent by reducing neural responses and hyperalgesic activities in an *in vivo* model of neuropathic pain [91]. Studies have also advocated the neuroprotective properties of *C. wightii* by modulating various markers of oxidative stress such as thiobarbituric acid reactive substances, nitric oxide (NO), TNF-α, glutathione (GSH), superoxide dismutase (SOD), and catalase levels [92], thus indicating its potential against neuroinflammation-related disorders [93]. The anticancer property of *C. wightii* has also been highlighted in several studies where it was found to inhibit cancer cell proliferation by inducing cell cycle arrest and apoptosis in prostate cancer (PC) cells [94, 95]. Similar anticancer activities were also reported by different compounds isolated from *C. wightii* in various cancers such as breast cancer, lung cancer, colon cancer, and melanoma [96]. Furthermore, *C. wightii* was shown to induce anti-bacterial properties by inhibiting the growth of gram-positive and gram-negative bacteria [97]. Additional findings also suggest the use of this plant and its extracts against gastric ulcer, skin injury [98], dementia [99], arthritic inflammation [100], and blood clots [101].

## Chemical nature of GS

**Figure 2.**
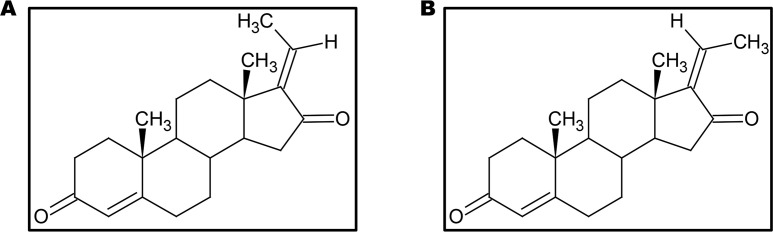
Structures of the isomers of GS. (A) E-GS; (B) Z-GS

GS, also known as 4, 17(20)-pregnadiene-3, 16-dione (C_21_H_28_O_2_), is the key component of the guggulu resin from *C. wightii*, which exists in E- and Z-isomers (Figure 2) and are represented as its *cis*- and *trans*-forms respectively [72, 77, 102, 103]. These E- and Z-GS are steroidal isomers and are inter-convertible in 3D space. They differ in the arrangement of CH3 molecule at C20 position and a distorted rotation of C-C double bond present at C17 and C20 positions is observed [104].

## Molecular targets of GS

**Figure 3.**
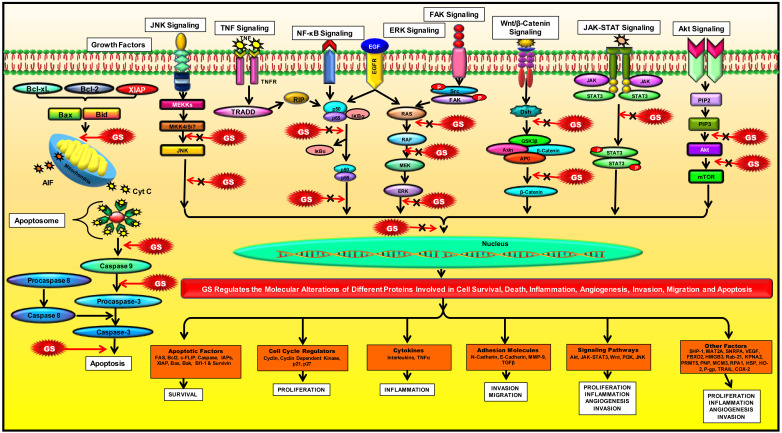
Modulation of molecular pathways by GS. GS modulates several signaling pathways and regulate the expression of various proteins involved in inflammation, apoptosis, cell cycle, angiogenesis, invasion, metastases, and chemoresistance

The multi-targeted compound, GS, is basically identified as an FXR inhibitor. FXR is a nuclear hormone receptor that regulates bile acid production and transport [79]. This protein might also plausibly regulate apoptosis in the cancer cells [105]. The activation of FXR is known to promote TGF-β-induced epithelial-mesenchymal transition (EMT), which is a hallmark of cancer [106]. FXR has also been identified as a marker of breast cancer-associated bone metastasis, and Z-GS has been found to efficiently inhibit FXR and its target associated bone proteins such as osteopontin, osteocalcin, and bone sialoprotein [107]. Besides FXR, GS is also known to alter the expression of various proteins in the cell and thus regulate different cellular processes such as cell growth, cell metabolism, cell survival, invasion, EMT and metastasis. Numerous preclinical studies on different cancer models have reported the chemoprotective effect of GS via modulation of multiple factors that are involved in cell growth and proliferation: COX-2, receptor activator of nuclear factor-κB ligand (RANKL); cell cycle proteins: c-Myc, cyclin D, cdc2, p21, p27; VEGF, MMP-9; proteins contributing to EMT: N-cadherin, TGF-β; molecules involved in apoptosis: caspases, survivin, Bcl-2, IAP1, XIAP, Bfl-1/A1, Bcl-2-associated X protein (Bax) and Bcl-2 homologous antagonist/killer (Bak); enzymes: IκBα kinase, Src homology 2 domain-containing protein tyrosine phosphatase 1 (SHP-1); markers involved in cell development: homeobox 2, caudal type homeobox 2 (CDX2) [65, 94, 106, 108–122]. Most of these targets constitute the key components of the major pathways regulated by GS in cancer cells such as the NF-κB pathway [123, 124]; the intrinsic mitochondrial apoptotic pathway [125, 126]; the JAK/STAT pathway [67] and the STAT3 pathway [123]. GS also regulates other pathways like the Akt pathway [66, 112]; the c-Jun N-terminal kinase (JNK) pathway [112, 127]; the extracellular signal-regulated kinase (ERK) pathway [127]; steroid Src/FAK signaling [67]; β-catenin signaling [128]; and the mitogen-activated protein kinase (MAPK) pathway [124]. Further, the proteomic profiling of GS treated cancer cells have revealed that GS-induced downregulation of several factors such as proteins contributing to cell growth: methionine adenosyltransferase 2A (MAT2A) and U1 small nuclear ribonucleoprotein A (SNRPA); proteins involved in cell growth and migration: F-box only protein 2 (FBXO2), high mobility group box 3 (HMGB3), and Ras-related protein Rab-21; proteins contributing to tumorigenesis: caveolin-1, importin karyopherin subunit alpha 2 (KPNA2), and protein arginine N-methyltransferase 5 (PRMT5); proteins involved in purine metabolism: purine nucleoside phosphorylase (PNP); proteins involved in DNA replication: DNA replication licensing factor minichromosome maintenance complex component 3 (MCM3), and replication protein A 70 kDa DNA-binding subunit (RPA1); heat shock proteins (HSP): heme oxygenase-2 (HO-2), Hsp70 and Hsp27 [126]. GS also induced upregulation of annexin A7, which is involved in exocytosis/tumor suppression and the cytoskeletal protein, filamin B that regulates cell shape and migration [126]. In concordance with other studies, this study also demonstrated that GS-mediated suppression of colorectal cancer (CRC) involved modulation of the prime cellular pathway, the TNF-α/NF-κB signaling pathway [126]. GS is also known to induce reactive oxygen species (ROS)-dependent apoptosis in cancer cells via modulation of JNK [129–131]. Furthermore, 14-3-3 zeta, which is involved in cancer recurrence and therapeutic resistance, has also been identified as a molecular target of GS [132, 133]. In addition, GS is also known to inhibit expression of P-glycoprotein (P-gp) in cancer cells, thereby chemosensitizing these cells to standard chemotherapeutic drugs [134–136]. For example, GS sensitized hepatocellular carcinoma (HCC) cells to doxorubicin (DOX) by modulation of COX-2/P-gp dependent pathway [137], MCF-7/DOX cells to DOX by reducing the levels of Bcl-2 and P-gp [138], gall bladder cancer cells to gemcitabine through suppression of NF-κB [139], and glioblastoma cells to sonic hedgehog inhibitor SANT-1 via inflection of Ras/NF-κB pathway [140]. Furthermore, the combination of GS with bexarotene decreased DOX resistance in breast cancer cells by stimulating the secretion of exosome-associated breast cancer resistance protein (BCRP) [141]. GS has also been reported to induce CCAAT/enhancer-binding protein homologous protein (CHOP)-dependent death receptor (DR)-5 expressions through modulation of ROS-dependent endoplasmic reticulum (ER)-stress, and thus sensitize liver cancer cells to TNF-related apoptosis inducing ligand (TRAIL)-induced apoptosis [142]. Thus, GS acts on a diverse range of molecular targets and prevent the development and progression of cancer (Figure 3).

The tumor microenvironment (TME) comprises of cancer cells, cancer stem cells, immune cells, factors such as growth factors, cytokines, enzymes, chemotherapeutic drugs, the components of the extracellular matrix (ECM), fibroblasts, inflammatory cells, blood vessels, and signaling molecules [143–146]. These components regulate tumorigenesis and various associated processes, such as oxidative stress, EMT, metastasis and autophagy [147–153]. The TME is immunosuppressive; therefore, identification of new and effective compounds that would restore the immune response in tumor cells and inhibit the progression of tumors is critical for the prevention and treatment of cancer [154, 155]. The natural compound, GS, plays a potential role in remodeling of TME by regulating oxidative stress, autophagy, and expression of ECM proteins. For instance, GS was reported to protect PC12 cells from hydrogen peroxide-induced oxidative stress by decreasing the levels of extracellular lactate dehydrogenase, NO and ROS, and preventing the loss of mitochondrial membrane potential (ΔΨm) [156]. The antioxidant activity of GS was further evinced through GS-induced reduction of plasma trimethylamine-*N*-oxide expression and stimulation of Nrf2/HO-1 signaling that protects the cells from ROS-induced oxidative stress [157, 158]. GS, being an FXR antagonist, was also found to be involved in the regulation of autophagy [159, 160]. This compound has also been reportedly associated with FXR-mediated differentiation of bone marrow stromal cells into osteoblasts or adipocytes [161]. A particular study also evinced GS-induced modulation of TME components like TGFβ-induced EMT markers and COX-2 in inflammatory cells [162]. Furthermore, GS was reported to regulate the survival and stimulation of hepatic stellate cells through the regulation of NF-κB and apoptosis [114]. Thus, GS shows efficacy in the regulation of TME remodeling and exerts a significant anticancer effect.

## Biological activities of GS

Congregate evidence suggests that GS has enormous potential in the prevention and treatment of various chronic diseases in humans owing to its multi-targeted properties. These include Alzheimer’s disease, arthritis, asthma, cancer, dermatitis, diabetes, gingivitis, inflammatory bowel disease, infectious diseases, intestinal metaplasia, otitis media, respiratory diseases, pancreatitis and psoriasis [79]. However, in the current review, we focus on its anti-cancer properties.

## Effect of GS in different cancers

Surfeit number of preclinical evidences unveiled the cancer chemopreventive and therapeutic properties of GS against a wide range of cancers, including cancers of the brain, breast, colon, head and neck, liver, pancreas and prostate, and hematological malignancies like leukemia and lymphoma.

These studies proved that GS has enormous potential for both the prevention and treatment of different cancers (Table 1) and are briefly summarized below.

**Table 1.** Potential of GS and its derivatives in the prevention and treatment of cancer

**Cancer**		**Model**	**Combination**	**Mechanism**	**References**
Brain tumor	*In vitro*	rBMECs	-	↓P-gp, ↑P-gp ATPase	[134]
	*In vitro*	A172, U87MG, T98G	SANT-1	↑Caspase-3, -9, ↑cytochrome c, ↑Bax, ↑IκBα, ↓pIκKα/β, ↓NF-κB, ↓Ras, ↓p-STAT3, ↑ERK, ↓c-Myc	[140]
Breast cancer	*In vitro*	MCF-7	IR	↓NF-κB, ↓ERα, ↓IGF-1Rβ, ↑p21 ↑Radiosensitivity	[172]
	*In vitro*	MCF-7/DOX	DOX	↑Apoptosis, ↓drug-transport activity, ↓MRP1	[174]
	*In vitro*	MDA-MB-231, MCF-7	-	↑Apoptosis, ↑Caspase-3, ↓β-Catenin, ↓TCF, ↓c-Myc, ↓Cyclin D1, ↓survivin	[128]
	*In vitro*	MCF-7	-	↓MMP-9, ↓p65/p50, ↓IκBα, ↓IKKαβ, ↓IKK/IκB/NF-κB axis^A^	[117]
	*In vitro*	MCF-7	-	↓MMP-9, ↓AP-1, ↓MAPK, ↓ERK, ↓JNK^B^	[117]
	*In vivo*	BALB/c mice	DOX	↓Tumor growth, ↓Bcl-2, ↓P-gp	[138]
	*In vitro*	MDA-MB-231	Bex & DOX	↑Apoptosis, ↓BCRP, ↓MDR proteins	[141]
CRC	*In vitro*	HT-29	-	↓STAT3, ↓ARNT, ↓VEGF, ↓MMP-2, -9	[110]
	*In vitro*	HT-29	-	↓Procaspase-9, -3, ↓Bcl-2, ↓cIAP-1 ↑Fas, ↑caspase-8, ↑p-JNK, ↑p-cJun, ↑tBid	[72]
	*In vivo*	nu/nu mice	-	↓Tumor growth	[72]
	*In vitro*	HT-29	IR	↓IGF-1Rβ, ↓NF-κB, ↑p21, ↑γH2AX	[172]
	*In vitro*	H508, SNU-C4, HT-29	-	↓FXR, ↑EGFR, ↑Src, ↑ERK-1/2	[177]
	*In vitro*	HCT-116	-	↑p-53, ↓NF-κB, ↓Bcl-2, ↓cIAP-1, ↓survivin	[126]
CCA	*In vitro*	Sk-ChA-1, Mz-ChA-1	-	↑Caspase-9, -3, -8, ↑PARP, ↓Bcl-2, ↓survivin	[111]
	*In vitro*	HuCC-T1, RBE	-	↑Caspase-9, -3, -8, ↑DR5, ↑tBid, ↓Bid, ↑p-JNK1/2, ↑p-p38, ↑p-ERK1/2, ↑P-eIF2α, ↑BiP, ↑GRP78, ↑CHOP	[130]
Esophageal cancer	*In vitro*	BE-derived cells	-	↑Apoptosis, ↑Caspase-3	[105]
	*In vitro*	Bic-1	-	↓CDX2	[113]
	*In vitro*	TE-3	-	↓Cell proliferation	[179]
		TE-12, SKGT-4, SKGT-5	-	↓FXR, ↓RAR-β2, ↓COX-2, ↓MMP-9, ↑Caspase-9, -3, -8	
	*In vivo*	nu/nu mice	-	↓Tumor growth	[179]
	*In vitro*	SKGT-4, SKGT-5, TE-3, TE-12	Amiloride	↓Cell viability, ↑apoptosis	[180]
	*In vivo*	nu/nu nude mice	Amiloride	↓Tumor formation, ↓growth	[180]
	*In vitro*	OE33, OE19	-	↓IκBα, ↓COX-2, ↓CDX-2, ↓PGE2	[115]
GBC	*In vitro*	TGBC1, TGBC2	-	↓NF-κB p65, ↓MMP-2, ↓VEGF-C	[139]
	*In vitro*	TGBC1, TGBC2	Gemcitabine	↓NF-κB p65	[139]
Haematological malignancies	*In vitro*	KBM-5	-	↓NF-κB, ↑Caspase, ↑PARP cleavage ↑TNF-induced apoptosis	[65]
	*In vitro*	U937	-	↓ΔΨm, ↓p-ERK, ↑ROS, ↑HO-1, ↓GSH	[202]
	*In vitro*	U937	-	↓Cyclin D1, ↓cdc2, ↓Bfl-1, ↓XIAP, ↓cFLIP, ↓survivin, ↓Bcl-xL, ↓Bcl-2, ↓COX-2, ↓c-Myc, ↓IL-6, ↓IL-1β, ↓TNF,↑p21, ↑p27, ↑caspase-8, -9, -3,↑PARP, ↑BiD, ↑Bax, ↑cytochrome c, ↑JNK, ↓PI3K/Akt	[112]
	*In vitro*	U266	-	↓STAT3, ↓c-Src, ↓p-JAK2, ↑SHP-1, ↓Bcl-2, ↓Bcl-xL, ↓Mcl-1, ↓cyclin D1, ↑Caspase-3, ↑PARP	[109]
	*In vitro*	MM.1S	-	↓STAT3	[109]
	*In vitro*	K562/DOX	-	↓P-gp, ↓MDR	[135]
HCC	*In vitro*	Hep3B	-	↑CHOP, ↑DR5, ↑ROS, ↑BiP, ↑p-IRE1, ↑p-JNK, ↑p-PERK, ↑eIF-2α, ↑ATF4	[142]
	*In vitro*	Hep3B	TRAIL	↓mtTMPt, ↑ Caspase-9, -3, -8, ↑PARP, ↓Bcl-2	[142]
	*In vitro*	HepG2	-	↓Bcl-2, ↑Bax, ↓TGF-β1, ↓VEGF, ↑TNF-α	[125]
	*In vitro*	HepG2R	-	↓COX-2, ↓P-gp	[137]
	*In vitro*	PLC/PRF/5R	DOX	↓COX-2, ↓P-gp, ↓PGE2, ↓MDR	[137]
	*In vitro*	HuH-7	-	↓EMT, ↓NR0B2, ↓CDH2 (N-cadherin)	[106]
HNC	*In vitro*	SCC4	-	↓STAT3	[109]
	*In vitro*	PCI-37a, UM-22b, 1483	-	↓STAT-3, ↓HIF-1α	[108]
	*In vivo*	Nude mice	-	↑Apoptosis, ↓STAT-3	[108]
	*In vitro*	SCC4	-	↓Cyclin-D1, ↓XIAP, ↓Mcl-1, ↓c-Myc, ↓Survivin, ↑caspase-9, -3, -8, ↑Fas/CD95, ↑Bax/Bcl2,	[133]
	*In vitro*	SCC4, HSC2	-	↓PI3K/Akt, ↓GSK3β, ↓PDK1, ↓p-Raf, ↓pS6, ↓p-Bax, ↓p-Bad	[66]
	*In vitro*	SCC4, HSC2	-	↓p-IκBα, ↓NF-κB p65, ↓COX-2, ↓IL-6, ↓p-STAT-3, ↓VEGF	[123]
Lung cancer	*In vitro*	H1299	-	↓NF-κB, ↓IκBα, ↓IKK, ↓COX-2, ↓MMP-9, ↓VEGF, ↓Cyclin D1, ↓c-Myc, ↓cIAP1, ↓XIAP, ↓Bfl-1, ↓Bcl-2, ↓TRAF1, ↓cFLIP, ↓survivin	[65]
Pancreatic cancer	*In vitro*	MIAPaCa-2, Panc-1	Gemcitabine	↓Bcl-2, ↓p-Akt, ↓NF-κB, ↑Bax, ↑p-JNK	[211]
	*In vivo*	BALB/c Nude Mice	Gemcitabine	↓NF-κB, ↓Akt, ↓Bcl-2, ↑JNK	[211]
	*In vitro*	CD18/HPAF, Capan1	-	↓XIAP, ↓Bcl2, ↓Cyclin D1, ↑BAD, ↑Bax, ↓MUC4, ↑Caspase-3, ↓JAK/STAT, ↓Src/FAK	[67]
	*In vitro*	MIAPaCa2, PANC-1	-	↓FXR	[209]
	*In vitro*	PC-sw	IR	↓IGF-1Rβ, ↓NF-κB	[172]
	*In vitro*	PANC-1	-	↓Akt^c^	[210]
PC	*In vitro*	PC-3	-	↓Bcl-2, ↓Bcl-xL, ↑Bax, ↑Bak, ↑caspase-9, -3, -8	[94]
	*In vitro*	PC-3, LNCaP	-	↑JNK1/2, ↑p38 MAPK, ↑ERK1/2	[131]
	*In vitro*	DU145, HUVEC	-	↓VEGF, ↓G-CSF, ↓IL-17, ↓MMP-2, ↓p-Akt, ↓VEGF-R2,	[218]
	*In vivo*	DU145 cells implanted nude mice	-	↓VEGF-R2, ↓factor VIII, ↓CD31	[218]
Skin cancer	*In vivo*	SENCAR mice	-	↓Skin edema, ↓hyperplasia, ↓ODC activity, ↓COX-2, ↓iNOS, ↓MAPKs, ↓NF-κB, ↓IKKα, ↓IκBα	[124]
	*In vitro*	B16/F10 mouse melanoma	-	↓Melanogenesis, ↓tyrosinase, ↓TRP-1 ↓TRP-2, ↓MITF	[219]

^A^ *Cis*-GS; ^B^ *Trans*-GS; ^C^ GS derivatives, GSD1 & GSD7; AP-1: activator protein 1; Bex: Bexarotene; BiP: binding immunoglobulin protein; cIAP-1: cellular inhibitor of apoptosis protein 1; HIF-1α: hypoxia-inducible factor 1alpha; IL-6: interleukin-6; iNOS: inducible nitric oxide synthase; MM: multiple myeloma; MMP-2: matrix metalloproteinase-2; mtTMPt: mitochondrial transmembrane potential; ODC: ornithine decarboxylase; STAT-3: signal transducer and activator of transcription 3; tBid: truncated Bid

### Brain cancer

Brain cancer consists of more than 120 types that cover 2% of the entire global cancer incidence. It can be defined as a primary and secondary tumor depending on its tumor development status, type of tissue, and nature of its malignancy [163, 164]. Few studies reported that GS exhibited significant anticancer properties against brain cancer cells in preclinical settings. For example, GS was shown to decrease the expression of P-gp and increase the activity of P-gp ATPase dose-dependently in rat brain microvessel endothelial cells (rBMECs), thus overcoming the low accumulation of the drug and resistance in brain cancer [134]. Furthermore, GS reduced expression of Ras and NF-κB in glioblastoma cells resulting in the sensitization of these cells to SANT-1 (which is a Gli1 protein inhibitor). It also increased the expression of caspases-3 and -9, Bax, and cytochrome c, which ultimately leads to the intrinsic pathway of apoptosis [140].

### Breast cancer

Breast cancer is the most commonly diagnosed cancer among females, and triple-negative breast cancer is the most aggressive form which is more frequently diagnosed in younger females with poor prognosis and a high recurrence rate [165–171]. Metastasis of breast cancer cells is common which is mostly seen in the bone, liver, lungs, and brain tissues [149] and contributes to an increased mortality rate due to this disease [146]. Several studies have well-documented the prospective of GS in inhibiting the growth of breast cancer. For example, a study showed that GS induced radiosensitization in breast cancer cells and reduced the growth of estrogen-positive tumors resistant to tamoxifen, through the suppression of NF-κB activation and IGF1-Rβ, and ERα [172]. Another study on the effect of GS isomers against breast cancer cells demonstrated that cis-GS repressed TPA-induced MMP expression by blocking IKK/NF-κB signaling, whereas trans-GS blocked the MAPK/AP-1 signaling. Moreover, the combination of these isomers exhibited an additive effect on the inhibition of invasion of MCF-7 cells [117]. Besides, the treatment of monocytes with GS reduced RANKL-activated NF-κB activation, which correlated with inhibition of IKK and phosphorylation and degradation of IκBα. In addition, GS also suppressed the differentiation of monocytes to osteoclasts that were induced by co-incubating breast cancer cells, MDA-MB-468, or human multiple myeloma cells, U266 with monocytes [116]. A study conducted by Silva et al. [173], revealed that the release of deoxycholate from osteoblast-like cells, MG63 and bone tissues trigger the survival and migration of metastatic breast cancer cells, MDA-MB231, which was inhibited upon treatment with GS through the induction of apoptosis and modulation of the expression of urokinase-type plasminogen activator (uPA).

Further, the guggulipid that contains GS has shown immense potential in the reduction of breast cancer cell growth and stimulation of apoptosis through the induction of cytoplasmic histone-associated DNA fragmentation, activation of caspase-3, and suppression of T-cell factor 4 and Wnt/β-Catenin pathway via suppression of its targets proteins such as cyclin D1, c-Myc, and survivin [128]. Additionally, the combined treatment of GS and DOX increased the sensitivity of the MCF-7/DOX cells to DOX by increasing the population of the apoptotic cells compared to DOX. This effect was plausibly mediated via the suppression of multi-drug resistance (MDR) protein, MRP1 [174]. Further, the combination of GS and DOX also inhibited tumor growth in BALB/c mice model by suppressing the expression of Bcl-2 and P-gp [138]. Similarly, the combination of GS and bexarotene increased DOX retention in breast cancer cells and enhanced cell death through increased secretion of exosome-associated BCRP/ABCG2 and reduced MDR levels [141].

### CRC

CRC is the third most common cancer occurring globally [175, 176]. Copious investigations have found the enormous potential of GS in combating this disease. For example, An et al. [72], found that treatment of HT29 colon cancer cells with GS elevated apoptosis by activating caspases-3 and -8. It has also been reported that GS increased the expression of truncated BH3 interacting domain death agonist (Bid), Fas, p-JNK, and p-c-Jun levels *in vitro* and decreased the expression of cIAP-1, cIAP-2, and Bcl-2, and suppressed tumor growth in an *in vivo* mouse model. Further, GS induced apoptosome and apoptosis in HCT 116 colon cancer cells via activation of caspase-3/7, through modulating the expression of Bcl-2, and releasing cytochrome c from mitochondria. The same study also showed that GS increased the levels of p53, and suppressed the expression of NF-κB and its regulated molecules, survivin, Bcl-2, and cIAP-1 [126]. Moreover, GS suppressed viability, angiogenesis, and metastasis of colon cancer cells by inhibiting the expression of VEGF, aryl hydrocarbon receptor nuclear translocator (ARNT), STAT3 proteins, and the activity of MMP-2 and -9 [110]. Besides, GS also increased the radiation sensitivity of colon cancer cells through the suppression of IGF1-Rβ and NF-κB [172]. Contrasting to other studies, Peng et al. [177], reported that over-expression of FXR suppressed the proliferation of H508, SNU-C4, and HT-29 colon cancer cells, whereas the inhibition of FXR by GS resulted in increased proliferation of the colon cancer cells through the upregulation of Src, EGFR, and ERK-1/2.

### Esophageal cancer (EC)

EC ranks eighth among all cancers globally and it is the sixth most common cause of cancer-related mortality with a 5-year survival rate of 15–25%. It originates in the GE junction and cardia and represents two major histological subtypes, i.e. esophageal squamous cell carcinoma and esophageal adenocarcinoma (EAC) [178]. Many studies have proven the efficacy of GS against EC. It was reported that overexpression of FXR in EAC is linked with increased tumor grade, tumor size, and high lymph node metastasis and inverse expression of retinoic acid receptor-β2 (RAR-β_2_), therefore knockdown of FXR yielded in decreased cell growth *in vitro* and decreased tumor size in the mouse model. This study suggests that GS holds enormous potential in the treatment of EC through the reduction of cell viability and induction of apoptosis via FXR suppression [179]. Similarly, the treatment of FXR overexpressed Barrett’s esophagus (BE)-derived cells with GS induced caspase-3 activity and resulted in apoptosis [105]. Further, GS treatment blocked IκBα phosphorylation induced by deoxycholic acid and decreased CDX2, COX-2, and prostaglandin E2 (PGE2) expressions in EAC cells, and also caused increased sub-G1 phase apoptosis in BE and EAC cells [115]. Besides, GS in combination with amiloride suppressed the viability of EC cells, induced apoptosis, and inhibited tumor growth in a mouse model [180].

### Head and neck cancer (HNC)

HNC includes a range of cancers among which more than 90% is represented by head and neck squamous cell carcinoma (HNSCC) [181–187]. HNSCC has a widespread global incidence rate with poor prognosis and a 50% occurrence in the oropharynx region mostly observed in the palatine tonsil and bottom of the tongue [188]. Several studies have demonstrated the potential of GS in inhibiting HNC. For instance, the SCC4 cells treated with GS were found to exhibit apoptosis via decreased expression of cyclin D1, phosphorylated Bcl-2-associated death promoter (BAD), XIAP, myeloid cell leukemia 1 (Mcl-1), c-Myc, and survivin, and increased expression of p21 and p27, Bax/Bcl2 ratio, caspases-9, -3, -8 and Fas/CD95 proteins [133]. Further, the pre-treatment of HNC cells with GS resulted in inhibition of smokeless tobacco/nicotine-induced expression of PI3K, phosphoinositide-dependent kinase-1 (PDK1), Akt, Raf, Glycogen synthase kinase 3 beta (GSK3β), and pS6, thereby blocking the PI3K/Akt pathway. It also suppressed Akt-associated Bax and BAD phosphorylation in HNC cells [66]. Similarly, in another study, GS was shown to suppress NF-κB and pSTAT3 and their target proteins COX-2 and VEGF, and also reduced the expression of phosphorylated IκBα and IL-6, induced by smokeless tobacco/nicotine in HNC cells [123]. Further, GS inhibited the growth of HNC cells by decreasing the expression of STAT3 [109]. The inhibition of STAT3 by GS was enhanced when combined with erlotinib or cetuximab *in vitro* and *in vivo*. Besides, GS also reduced the invasion of HNC cells by suppressing the expression of HIF-1α [108]. Additionally, it was also shown that the proteasome inhibitor bortezomib causes an increase in total STAT3, pSTAT3, and cellular STAT3 levels in HNC cells and the combined treatment of GS (natural STAT3 inhibitor) with bortezomib could induce synergistic death of cancer cells [189].

### HCC

HCC or liver cancer is one of the highest occurring malignancies in the world [2, 53, 190–197]. Despite the advancement in therapeutic strategies, there is a lack of efficacious and non-toxic drugs for the treatment of HCC. In pursuit of alternative medicine for the treatment of HCC, GS was investigated in several studies. For instance, treatment with GS significantly reduced the proliferation, induced G0/G1 phase arrest and apoptosis in HepG2 cells via regulating the expression of Bax, Bcl-2, TGF-β1, TNF-α, and VEGF [125]. Further, GS was also found to inhibit TGF-β-induced EMT in HCC cells [106]. In this study, GS was found to decrease the mRNA expression of *CDH2*, which codes for N-cadherin, a marker of EMT, and nuclear receptor subfamily 0, group B, member 2 (*NR0B2*), which is an *FXR* target gene [106]. Additionally, the treatment of GS sensitized DOX-resistant PLC/PRF/5R cells to DOX via suppression of the expression of COX-2, P-gp, and PGE2 [137]. In another study, the combination of GS with TRAIL was found to induce apoptosis in HCC cells through the reduction of mitochondrial transmembrane potential and caspase activation [142]. The receptor for TRAIL, DR5, was also found to be upregulated along with activation of proteins associated with ER stress and apoptosis such as eukaryotic initiation factor-2α (eIF2α) and CHOP in GS treated cells [142].

### Hematological malignancies

Hematological malignancies such as leukemia, lymphoma, and multiple myeloma are serious health issues worldwide [2, 198–201]. Most of the hematological malignancies are a result of genetic alterations, as an example, the mutation of FMS-like tyrosine kinase3-internal tandem duplication (FLT3-ITD), which is common in acute myeloid leukemia (AML) [27, 29]. Therefore, the therapeutic potential of GS was explored for the treatment of hematological malignancies. GS was shown to potentiate the TNF-induced apoptosis of leukemia cells by inhibiting NF-κB and increasing caspase-mediated cleavage of PARP protein [65]. This plant sterol was also reported to reduce the proliferation of U937 leukemia cells by inhibiting DNA synthesis, inducing G1/S phase arrest, and decreasing the levels of cyclin D1 and cdc2 and upregulating CDK inhibitors such as p21 and p27. Further, GS was shown to induce apoptosis in these cells through activation of caspases-3, -8, and -9, the release of cytochrome c, Bid and PARP cleavage, and by decreasing anti-apoptotic proteins such as Bfl-1, XIAP, cFLIP, Bcl-xL, Bcl-2, survivin, COX-2, c-Myc, IL-6, IL-1β and TNF levels. In addition, GS also activated JNK and suppressed Akt activity in these cells [112]. Moreover, the *cis*- and *trans-*GS was reported to cause apoptosis in AML cells, HL60 and U937, through phosphatidylserine externalization and loss of ΔΨm. The trans-GS was also reported to play a remarkable role in the differentiation of these cells as evidenced through elevated levels of surface proteins such as CD11b and CD14, while *cis*-GS reduced intracellular GSH levels and promoted oxidation of cardiolipin, which is known to be involved in mitochondrial function and prevention of apoptosis [202]. Moreover, the combined treatment of GS and DOX reversed MDR by inhibiting P-gp and increasing cellular accumulation of DOX in K562/DOX cells [135]. Additionally, GS inhibited the proliferation of multiple myeloma cells by decreasing the expression of STAT3 via activation of SHP-1, which leads to the suppression of c-Src and p-JAK2 proteins. Further, GS was also shown to inhibit *Bcl-2*, *Bcl-xL*, *Mcl-1*, and *cyclin D1* gene expressions in these cells [109].

### Pancreatic cancer (PaCa)

PaCa is one of the most fatal malignancies in the world that ranks seventh in both males and females worldwide [2]. Studies have shown the efficacy of natural compounds like resveratrol, curcumin, γ-tocotrienol, food supplement like Zyflamend^TM^, and small molecules like protein kinase D (PKD) inhibitor, CRT0066101 against PaCa [203–208]. Similarly, GS has also been reported to exhibit antineoplastic and chemosensitizing potential in PaCa models. In a particular study, GS was found to suppress proliferation and survival and induce apoptosis in PaCa cells, Capan1 and CD18/HPAF, through enhanced activation of caspase-3, altered BAD phosphorylation, decreased cyclin D1, and reduced level of anti-apoptotic proteins, Bcl-2 and XIAP. This compound also suppressed invasion and metastasis in PaCa cells by dysregulating the cytoskeletal organization, inhibiting the activation of FAK and Src signaling, and reducing MMP-9 levels and JAK/STAT pathway-mediated mucin4 (*MUC4*) expressions [67]. Similarly, GS-mediated FXR inhibition was found to remarkably suppress migration and invasion in PaCa cells [209]. Besides, the derivatives of GS, GSD-1, and GSD-7 were also reported to induce morphological changes and reduce cell survival in PANC-1 cells by inhibiting Akt protein [210]. Further, GS was also evinced to induce radiosensitization in PC-Sw cells through reduced levels of NF-κB and IGF1-Rβ [172]. In addition, GS administration enhanced gemcitabine-mediated growth suppression and apoptosis in PaCa cells via suppression of NF-κB activity, levels of Akt and Bcl-2, and activation of c-JNK and Bax [211].

### PC

In terms of incidence, PC is the second most common malignancy in males worldwide [2, 212–215]. The factors like unhealthy diet and lifestyles were reported to be associated with the development of PC [216, 217]. Multiple lines of evidence indicate that GS has immense potential in the prevention and treatment of PC. For example, GS induced apoptosis in PC-3 and LNCaP cells by triggering ROI-dependent JNK activation [131]. In LNCaP cells, GS was also found to reduce the expression and promoter activity of the androgen receptor [131]. Besides, GS treatment was also reported to induce apoptosis in PC-3 cells by inducing DNA fragmentation and upregulating the expression of Bax, Bak, and caspases -3, -8, and -9 [94]. Further, the administration of Z-GS was reported to inhibit tube formation of HUVEC cells and migration of DU145 and HUVEC cells via suppression of VEGF, granulocyte colony-stimulating factor (G-CSF), VEGF receptor (VEGF-R2), and Akt. The anti-angiogenic effect of GS was also evident in the nude mice model, where the oral administration of GS was found to suppress tumor growth and decrease levels of angiogenic markers, factor VIII, CD31, and VEGF-R2 [218].

### Other cancers

Apart from the aforementioned cancers, the anticancer potential of GS was documented in other cancers such as cholangiocarcinoma (CCA), gallbladder cancer, melanoma, and lung cancer. For example, GS was shown to reduce the growth of CCA cells (Sk-ChA-1 and Mz-ChA-1) and induce apoptosis via enhancement of caspases -3, -8, and -9 expression and PARP cleavage, and suppression of survivin and Bcl-2 [111]. Further, GS treatment was reported to induce apoptosis in HuCC-T1 and RBE CCA cells through the regulation of the ROS/JNK pathway [130]. In addition, treatment with GS was shown to inhibit melanogenesis in B16 murine melanoma cells through the reduction of tyrosinase, microphthalmia-associated transcription factor (MITF), and tyrosinase-related protein (TRP-1 and TRP-2). This compound was also found to suppress melanogenesis induced by α-melanocyte-stimulating hormone and forskolin [219]. Another study revealed that GS lowered skin tumor incidence in SENCAR mice by inhibiting various inflammation and tumor-associated markers such as COX-2, iNOS, MAPKs, IKKα, IκBα, and NF-κB [124]. Furthermore, the activity of GS was also studied in H1299 lung cancer cells where it inhibited NF-κB activation induced by various agents such as TNF, IL-1β, and carcinogens; and also inhibited IκBα and IKK. Besides, GS suppressed the activities of other proteins such as COX2, MMP9, VEGF, cell cycle proteins, and anti-apoptotic proteins [65]. In another study, the potential of GS on gall bladder cancer (GBC) was investigated. This study showed that GS suppressed the proliferation and invasion of TGBC1 and TGBC2, GBC cells via inhibition of NF-κB p65, VEGF-C, and MMP-2. In addition, the combination of GS and gemcitabine significantly inhibited the growth of GBC, thereby exerting its chemosensitizing potential [139]. Thus, GS has immense potential as a drug candidate for cancer treatment as proved by preclinical studies. However, the pharmacokinetics (Pk) and pharmacodynamics of this compound should be studied for its safe and effective use as a clinical drug.

## Pk and pharmacodynamics of GS

A limited number of studies have reported the Pk and pharmacodynamics of GS to date. In 1998, Verma et al. [220], performed HPLC of Z-GS, E-GS, drug-free rat serum, GS-spiked serum, serum from GS (50 mg/kg)-dosed rats (at 4 h), and serum from GS-dosed rats (at 24 h). The recovery percentage of Z-GS and E-GS from spiked serum samples was more than 90% at all concentrations of spiking (25, 50, 250, 2, 500 ng/mL) while the chromatogram analysis suggested that under *in vivo* conditions, GS is plausibly metabolized from Z- to E-form upon administration and is retained in the same form in the body. In another study, the effect of both oral and intravenous administration on various Pk parameters of Z-GS and E-GS were analysed; however, no statistically significant difference was observed. It was reported that the Pk parameters like terminal half-life, systemic clearance, area under the curve, and volume of distribution were 4.48 and 3.56 h, 1.76 and 2.24 L/h, 5.95 and 4.75 μgh/mL, and 11.36 and 10.76 L, respectively. These results demonstrated that the absorption of Z-GS was rapid from the gastrointestinal tract, leading to maximum concentration (C_max_) in the serum 2 h after oral administration. Moreover, the bioavailability of orally administered Z-GS relative to the intravenous administration was found to be 42.9% [221]. In another study, Bhatta et al. [222], developed a liquid chromatography-tandem mass spectrometry (LC-MS/MS) method for the simultaneous determination of both Z- and E-GS in rabbit plasma. However, this method faced a limitation of the long run time of 20 min. Subsequently, in 2015, Chhonker et al. [223], developed and validated an extremely specific and sensitive LC-MS/MS method for the estimation of E- and Z-GS in rat plasma within a run time of 6 min. Using this method, the ADME properties of GS such as metabolic stability, pH-dependent stability, plasma protein binding, solubility and Pk of GS isomers administered orally in rats were evaluated. Moreover, E- and Z-GS were reported to be soluble up to 50 μM (1, 561 ng/mL) under physiological conditions. The findings also suggested that E- and Z-GS were stable in the gastrointestinal fluid and were not subjected to enzymatic degradation; their plasma protein binding was high and independent of the concentration of GS. Further, both the stereoisomers exhibited low bioavailability of GS owing to wide first pass metabolism through high clearance and short half-life in rats [223].

The efficacy, toxicity, and pharmacodynamics of a drug depend hugely on its metabolic fate. In 2012, Yang et al. [224], reported a basic metabolic profile of Z-GS. However, to gain an in-depth understanding of the Pk and metabolism of GS, in 2018, Chhonker et al. [225], conducted another metabolic investigation where GS was found to metabolize to produce nineteen metabolites in human liver microsomes, and S9 fractions and hydroxylation was identified as the prime metabolic pathway. Also, the binding efficiency of GS with human serum albumin ranged from low to moderate. Further, CYP profiling and inhibition studies revealed that GS is a substrate for various CYPs, majorly CYP3A4, and it inhibited CYP2C19 [225]. Recently, another study was performed to detect electrophilic reactive metabolites of GS isomers. It was hypothesized that these metabolites are responsible for the toxic reactions of GS. The results showed that hydroxylated metabolites of GS isomers formed adducts with the trapping agents, GSH and N-acetylcysteine [226]. Overall, these studies form the background for further structural modification of GS, enhancement in the stability, and designing of its analogs.

## Discussion and conclusion

The increasing rate of cancer cases and deaths pose a huge concern worldwide [227–231]. The currently available drugs are expensive, induce severe adverse side-effects, and are not very effective [6, 38, 232]. Thus, alternative drugs are sought for the efficient management of cancer, and natural compounds have shown promising results [233–245]. Among these compounds, GS, the FXR antagonist, has exhibited immense potential as an anticancer agent against various cancer types. This multi-targeted agent has shown to affect different cellular processes such as inflammation, cell proliferation, survival, angiogenesis, invasion, metastasis, EMT, and apoptosis in cancer cells in various preclinical models. In this process, GS has been reported to inflect multiple pathways like NF-κB, STAT-3, ERK/MAPK, JAK/STAT, ROS/JNK and PI3K/Akt, and several genes and proteins such as COX-2, MMP-9, p38, PGE2, HIF-1α, VEGF, interleukins, cyclin D1, survivin, p21, p27, p53, PARP, Bid and caspases. This active metabolite of guggulipid has also been known to regulate cellular stress and ΔΨm in several *in vitro* models. Further, GS also inhibited tumor growth remarkably in *in vivo* models of cancer. Not only this, but GS could also induce sensitization of cancer cells to standard chemotherapeutic drugs like DOX and gemcitabine in various cancer models. It has also been reported to sensitize the cancer cells to TRAIL and enhance the efficacy of radiation in cancer models, thereby leading to cancer cell death. These studies show that GS is a potential therapeutic agent for the prevention and treatment of various cancers. However, the preclinical findings need to be validated in clinical settings with a detailed investigation of its safety, toxicity, and bioavailability. Studies have identified two prime stereoisoforms of GS, Z-GS and E-GS, along with 19 metabolites. GS is characterized by physiological solubility upto 50 μM, high plasma protein binding, and limited bioavailability. Hence, studies have been carried out to design analogs of this pharmacophore with enhanced bioavailability. Thus, modulation of the pharmacodynamics and Pk properties of this compound may lead to the development of analogs and derivatives of GS for better management of cancer.

## Abbreviations

AML: acute myeloid leukemia
ARNT: aryl hydrocarbon receptor nuclear translocator
BAD: Bcl-2-associated death promoter
Bak: Bcl-2 homologous antagonist/killer
Bax: Bcl-2-associated X protein
Bcl-2: B-cell lymphoma 2
BCRP: breast cancer resistance protein
BE: Barrett’s esophagus
Bfl-1/A1: Bcl-2-related protein A1
Bid: BH3 interacting domain death agonist
*C. wightii*: *Commiphora wightii*
CCA: cholangiocarcinoma
CDX2: caudal type homeobox 2
cFLIP: cellular FLICE (FADD-like IL-1β-converting enzyme)-inhibitory protein
CHOP: CCAAT/enhancer-binding protein homologous protein
COX-2: cyclooxygenase 2
CRC: colorectal cancer
DOX: doxorubicin
DR: death receptor
EAC: esophageal adenocarcinoma
EC: esophageal cancer
ECM: extracellular matrix
eIF2α: eukaryotic initiation factor-2α
EMT: epithelial-mesenchymal transition
ER: endoplasmic reticulum
ERK: extracellular signal-regulated kinase
FAK: focal adhesion kinase
FXR: farnesoid X receptor
GBC: gall bladder cancer
GS: guggulsterone
GSH: glutathione
GSK3β: Glycogen synthase kinase 3 beta
HCC: hepatocellular carcinoma
HNC: head and neck cancer
HNSCC: head and neck squamous cell carcinoma
IAP1: inhibitor of apoptosis protein 1
IKK: IkappaB kinase
IκBα: IkappaB alpha
JAK: Janus kinase
JNK: Jun N-terminal kinase
LC-MS/MS: liquid chromatography-tandem mass spectrometry
LDL: low-density lipoprotein
MAPK: mitogen-activated protein kinase
Mcl-1: myeloid cell leukemia 1
MDR: multi-drug resistance
MITF: microphthalmia-associated transcription factor
MMP: matrix metalloproteinase
MUC4: mucin 4
NF-κB: nuclear factor kappa B
NO: nitric oxide
NR0B2: nuclear receptor subfamily 0, group B, member 2
PaCa: pancreatic cancer
PC: prostate cancer
PDK1: phosphoinositide-dependent kinase-1
PGE2: prostaglandin E2
P-gp: P-glycoprotein
PI3K: phosphoinositide 3-kinase
Pk: pharmacokinetics
PKD: protein kinase D
PPARγ: peroxisome proliferator-activated receptor-gamma
QOL: quality of life
RANKL: receptor activator of nuclear factor-κB ligand
rBMECs: rat brain microvessel endothelial cells
ROS: reactive oxygen species
SHP-1: Src homology 2 domain-containing protein tyrosine phosphatase 1
Src: steroid receptor coactivator
STAT: signal transducers and activators of transcription
TGF-β: transforming growth factor β
TME: tumor microenvironment
TNF: tumor necrosis factor
TNFR: tumor necrosis factor receptor
TRAIL: TNF-related apoptosis-inducing ligand
TRP: tyrosinase-related protein
VEGF: vascular endothelial growth factor
XIAP: X-linked inhibitor of apoptosis protein
ΔΨm: mitochondrial membrane potential

## Declarations

### Copyright

© The Author(s) 2020.
